# Prolonged Elimination of Lamotrigine After Suicidal Overdose: A Case Report

**DOI:** 10.7759/cureus.78803

**Published:** 2025-02-10

**Authors:** Hiroshi Mae, Mizuho Namiki, Mitsuru Shiokawa, Munekazu Takeda, Shusuke Mori

**Affiliations:** 1 Department of Pharmacy, Tokyo Women's Medical University, Tokyo, JPN; 2 Department of Critical Care and Emergency Medicine, Tokyo Women's Medical University, Tokyo, JPN

**Keywords:** drug metabolism, lamotrigine, mental disorders, overdose, prolonged elimination

## Abstract

Lamotrigine (LTG) is an antiepileptic drug that stabilizes neuronal membranes by inhibiting excitatory neurotransmitter release, thereby reducing neuronal excitation. Various factors can contribute to its metabolic dysfunction. A 22-year-old female with bipolar disorder presented after intentionally ingesting 216 tablets, including LTG (4,100 mg), prescribed antipsychotics, over-the-counter cold medicine containing acetaminophen (27 g), and alcohol. She arrived at the hospital approximately one-hour post-ingestion in a confused state, with mild transaminase elevation but no significant electrocardiographic abnormalities. Initial management included activated charcoal, N-acetylcysteine therapy, and intravenous fluids. Despite these interventions, she deteriorated to a comatose state by hospital day two, accompanied by a mild skin rash later diagnosed as urticaria. Her hepatic function and consciousness gradually improved, with transaminase normalization and full neurological recovery by day six. She was discharged on day seven without residual deficits. Serial LTG serum measurements at 13, 55, and 79 hours post-ingestion (16.10 mg/L, 12.39 mg/L, and 6.84 mg/L, respectively) revealed a non-linear elimination pattern, with an estimated half-life of ~100 hours between the first two measurements. However, by the third measurement, elimination aligned with previously reported pharmacokinetics. The patient’s young age, normal BMI, absence of hepatic disease, and minimal interaction potential with co-ingested antipsychotics did not explain the delayed LTG clearance. We hypothesize that hepatic metabolic pathway saturation led to this non-linear pharmacokinetic behavior. The resolution of delayed elimination corresponded with hepatic enzyme normalization. In drug overdose cases, prolonged consciousness disturbance may indicate an extended drug half-life due to metabolic dysfunction. If recovery exceeds expectations, further evaluation is warranted to identify and address potential causes.

## Introduction

Lamotrigine (LTG) is an antiepileptic drug that stabilizes neuronal membranes by inhibiting voltage-sensitive sodium channels, thereby reducing the release of excitatory neurotransmitters such as glutamate. It is primarily used to manage epilepsy and bipolar disorder. While generally well-tolerated at therapeutic doses, LTG may occasionally cause adverse effects, including dizziness, somnolence, headache, anxiety, and hallucinations. Rare but severe dermatological reactions, such as Stevens-Johnson syndrome and toxic epidermal necrolysis, can occur, particularly at elevated serum concentrations. To minimize this risk, LTG is initiated at a low dose of 25 mg and gradually titrated to a maintenance dose of 200 mg under careful monitoring. LTG undergoes hepatic metabolism primarily through glucuronide conjugation, with subsequent renal excretion of inactive metabolites. Although routine therapeutic drug monitoring is not standardized, serum concentrations of 3-13 mg/L are frequently cited, with levels around 3 mg/L commonly observed in clinical practice following regular use [1].

Non-linear pharmacokinetics occurs when LTG elimination deviates from first-order kinetics, often due to enzyme saturation at high concentrations. This results in a disproportionate increase in plasma levels with increasing doses and an extended half-life, as seen in overdose cases. LTG typically exhibits linear pharmacokinetics, where its plasma concentration increases proportionally with the dose. However, certain conditions can lead to non-linear pharmacokinetics, resulting in disproportionate increases in plasma levels with higher doses. Factors such as weight, age, smoking, and co-therapy with enzyme inducers or inhibitors significantly affected LTG clearance. Notably, smoking increased LTG clearance by 34%, and co-administration with valproic acid and sertraline reduced LTG clearance by 58%. These findings suggest that metabolic saturation can occur under certain conditions, leading to non-linear pharmacokinetics [2].

Overdose cases involving LTG are well-documented, often resulting from intentional ingestion in adults or accidental ingestion in children. Most cases have benign outcomes; however, sensory disturbances (e.g., altered consciousness, seizures, or agitation) occur in approximately 50% of patients, while cardiac effects (e.g., arrhythmic tachycardia or prolonged QRS complexes) are reported in about 30%. Severe complications, including life-threatening arrhythmic tachycardia, cardiac arrest, complete heart block, multiple organ failure, or aspiration pneumonia, are rare but have been reported, with fatalities documented in some instances. Serum LTG concentrations in overdose scenarios typically peak between 25 and 75 mg/L, although the timing and ingested dose are often unclear. Additionally, higher reported concentrations may reflect publication biases [3].

Despite several case reports, comprehensive analyses of the time course of LTG serum concentrations following overdose, particularly those based on serial sampling at multiple time points, remain limited in the literature. This report presents a novel detailed analysis of the clinical presentation and temporal profile of LTG serum concentrations, derived from serial measurements taken at three distinct time points following a significant overdose, contributing new insights into the pharmacokinetics of LTG overdose management.

## Case presentation

A 22-year-old female with bipolar disorder presented to the emergency department after intentionally ingesting a large quantity of medications and alcohol. She contacted emergency services herself and was suspected to have consumed 216 prescription tablets, including 4,100 mg of LTG (25 mg/tab × 164 tabs), 262.5 mg of olanzapine (10 mg/tab and 2.5 mg/tab × 21 tabs), 10 mg of risperidone (1 mg/tab × 10 tabs), and 27 g of acetaminophen from 270 over-the-counter cold medication tablets.

The patient had been experiencing a persistent depressive state for nine months before presentation and was diagnosed with bipolar affective disorder at a nearby psychiatric clinic. Treatment was initiated with escitalopram; however, due to significant depressive symptoms, it was switched to LTG. Two months prior to the presentation, the patient experienced suicidal ideation and ingested 50 tablets of unspecified prescription medication, leading to emergency transport to another hospital, where she was admitted for one night. Subsequently, olanzapine was added to the regimen, resulting in clinical improvement. Other psychiatric medications included lorazepam, brotizolam, flunitrazepam, and risperidone.

Approximately one hour after ingestion, she arrived in a confused state (Japan Coma Scale (JCS) I-3). Vital signs revealed a temperature of 36.9 ℃, heart rate of 117 bpm, blood pressure of 148/116 mmHg, respiratory rate of 19 breaths/min, and oxygen saturation of 94% on room air. Her weight was 52 kg, and her height was 150 cm. Physical examination showed no skin rash. The blood test results are shown in Table 1. No elevation in lactate levels, no abnormalities in the complete blood count, no evidence of renal dysfunction, and mild hepatic impairment, characterized by an elevation in transaminases, were noted. Electrocardiography (ECG) showed no abnormalities, such as QRS widening or QT prolongation. A rapid urine drug screen was positive only for benzodiazepines.

**Table 1:** Laboratory findings on admission with a reference range Abbreviations: anion gap: AG, lactate: Lac, white blood cells: WBC, neutrophils: Neut, hemoglobin: Hb, platelets: Plt, total bilirubin: T-bil, aspartate aminotransferase: AST, alanine aminotransferase: ALT, blood urea nitrogen: BUN, serum creatinine: Cre, estimated glomerular filtration rate: E-GFR, sodium: Na+, potassium: K+, chloride: Cl-, total protein: TP, albumin: Alb, C-reactive protein: CRP

Items	Value	Reference range	Unit
Arterial Blood Gas			
pH	7.383	7.35-7.45	
pCO_2_	43	35-45	mmHg
HCO_3_^-^	25	22-26	mmol/L
AG	13	8-12	mEq/L
Lac	1.36	0.5-1.5	mmol/L
Complete Blood Count			
WBC	6.49	4.0-8.6	×10³/μL
Neut	54%	38-71	%
Hb	13.3	12.0-16.0	g/dL
Plt	40.5	15.0-35.0	×10⁴/μL
Biochemistry			
T-bil	0.5	0.2-1.2	mg/dL
AST	40	13-33	U/L
ALT	46	6-30	U/L
BUN	12.9	8.0-20.0	mg/dL
Cre	0.64	0.48-0.79	mg/dL
E-GFR	96.2	>90	mL/min/1.73 m²
Na^+^	142	135-145	mEq/L
K^+^	4	3.4-4.9	mEq/L
Cl^-^	108	98-108	mEq/L
TP	6.6	6.5-8.2	g/dL
Alb	4.5	3.8-5.1	g/dL
CRP	0.16	<0.33	mg/dL

**Figure 1:**
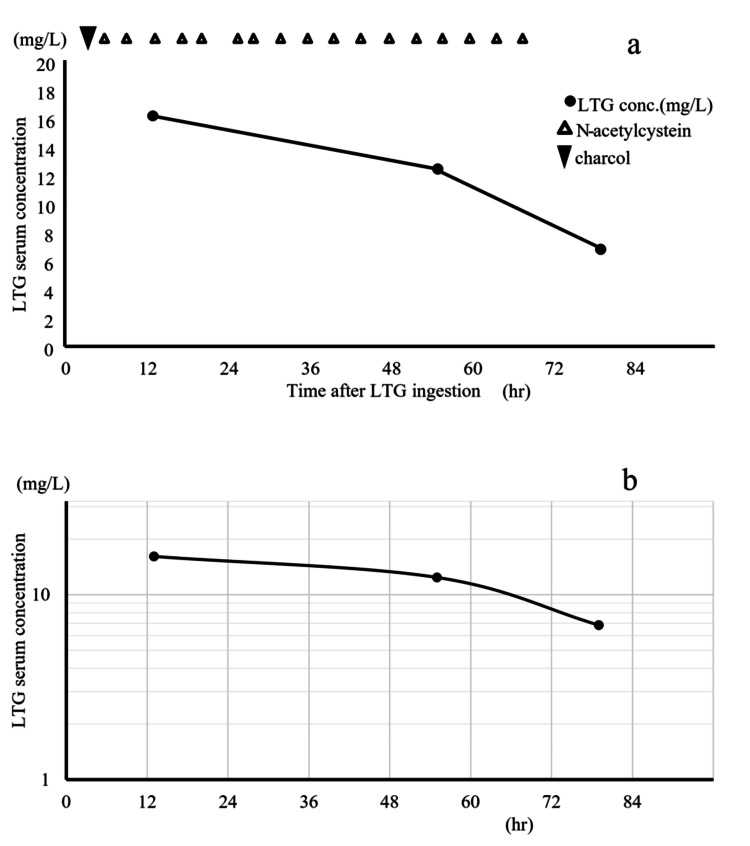
Therapies and changes in the serum concentrations of lamotrigine (LTG) a) This graph plots LTG serum concentration (mg/L) on the vertical axis against elapsed time (hours) on the horizontal axis. Serial measurements of LTG serum concentrations revealed levels of 16.10 mg/L, 12.39 mg/L, and 6.84 mg/L at 13, 55, and 79 hours post-ingestion, respectively. From this graph, by calculating the half-life of LTG between 13 hours and 55 hours, the formula y = -0.083333x + 17.24833 can be derived, from which the half-life can be determined. Since the half-life corresponds to the x-value when y equals 17.24833/2, the calculated half-life is 103.49 hours. In drugs that follow linear pharmacokinetics, the graph should theoretically be concave, based on the half-life theory. However, in this case, the pharmacokinetic graph is convex. This deviation from typical pharmacokinetics suggests delayed metabolism in the early stages, indicating that the drug follows a non-linear pharmacokinetic pattern. b) The graph is a logarithmic representation of LTG values taking into account metabolic time. The rate of decline in LTG levels between 55 and 79 hours post-ingestion is greater than that observed between 13 and 55 hours, indicating a prolonged metabolic phase of LTG during the initial stage. Regarding the logarithmic graph, drugs that follow linear pharmacokinetics should produce a straight line. However, the graph in this case is convex, indicating that the drug's pharmacokinetics in the early stage do not follow a typical pattern, suggesting delayed metabolism and a non-linear pharmacokinetic behavior. Abbreviation: LTG, lamotrigine

Management included the insertion of a nasogastric tube and detoxification with 50 g of activated charcoal, 34 g of magnesium citrate, and 75 mg of sodium picosulfate hydrate. Supportive care with fluid therapy was initiated, and to address acetaminophen toxicity, 7 g of N-acetylcysteine (NAC) was administered, followed by 3.5 g every four hours for 17 doses over a period of two to 65 hours after ingestion. Serial LTG serum concentrations were recorded as 16.10 mg/L, 12.39 mg/L, and 6.84 mg/L at 13, 55, and 79 hours after ingestion, respectively (Figure 1).

On day two, her condition worsened to a deep comatose state (JCS III-100). Gradual neurological improvement followed, with recovery to JCS II-10 on day three and JCS I-1 on day four. Liver transaminase levels remained within normal ranges throughout. A mild skin rash appeared on her chest, face, and lower extremities on day two, diagnosed as urticaria by a dermatologist. Antihistamines were prescribed, and the rash was monitored. No further treatment was performed, nor were any imaging studies, including a CT scan, performed. Another arterial blood gas test taken on day three showed no abnormalities, with a pH of 7.440, pCO₂ of 35.2 mmHg, and lactic acid of 0.8 mmol/L. The patient did not require supplemental oxygen, and fluid therapy was discontinued by day four. During the hospital stay, the psychiatric liaison team intervened and provided psychotherapy. As the patient’s consciousness fully recovered without functional impairments and her suicidal ideation resolved, she was discharged on day seven (Tables 2-3). A referral letter was issued to the primary care psychiatrist, requesting continued management. No modifications were made to the patient's existing medication regimen at the time of discharge.

**Table 2:** Clinical course after lamotrigine (LTG) overdose Mild hepatic dysfunction was noted initially, with transaminase levels progressively declining to within the normal range. While the patient’s level of consciousness worsened to III-100 on the second day, full recovery was achieved by the sixth day. Abbreviations: AST: aspartate aminotransferase, ALT: alanine aminotransferase, JCS: Japan Coma Scale, RA: room air, SR: sinus rhythm

Items	Day 1	Day 2	Day 3	Day 4	Day 5	Day 6	Day 7
AST (U/L)	40	30	23	21	17	19	-
ALT (U/L)	48	39	34	30	28	30	-
Conscious Level (JCS)	Ⅰ-3	Ⅲ-100	Ⅱ-10	Ⅱ-10	Ⅰ-1	0	0
SpO_2_(%) (Oxygen Therapy)	94 (RA)	95 (RA)	95 (RA)	94 (RA)	95 (RA)	96 (RA)	98 (RA)
ECG	SR	SR	SR	SR	SR	-	-
Event	-	Mild skin eruption	-	-	-	-	Discharge

**Table 3:** Japan Coma Scale (JCS) for grading impaired consciousness This table is a Japanese-to-English translation of the Japan Coma Scale (JCS), which is universally used in Japan. The JCS is the scale for assessing a patient’s consciousness level, consisting of zero, one, two, and three Roman numeral digital codes corresponding to alert, awake without stimuli, arousable with some stimuli (but reverts to previous status if stimulus stops), and unarousable with any forceful stimuli, respectively, which are further divided into sub-classes of 1-3, 10-30, and 100-300, respectively, to create a 10-degree scale between alert and comatose.

Grade	Consciousness Level
I	Awake (scored in single digits)
1	Fully conscious and alert
2	Disoriented
3	Unable to state their name or date of birth
II	Temporarily arousable with stimulation (scored in two digits)
10	Opens eyes with normal verbal stimulation
20	Opens eyes with loud verbal stimulation or vigorous shaking
30	Barely opens eyes with continued verbal stimulation and painful stimuli
III	Unresponsive to stimulation (scored in three digits)
100	Reacts to pain with defensive movements, such as swatting away
200	Moves limbs or grimaces in response to painful stimuli
300	No response to painful stimuli

## Discussion

LTG undergoes hepatic metabolism via glucuronidation, yielding inactive metabolites. This process is independent of the cytochrome P450 (CYP450) enzyme system. LTG serum concentrations exhibit linear pharmacokinetics, influenced by increased clearance with chronic administration due to autoinduction. Consequently, the elimination half-life is approximately 30-50 hours without evidence of non-linear metabolism at therapeutic doses [4].

LTG typically follows linear pharmacokinetics at therapeutic doses, where plasma concentration increases proportionally with the dose. However, at high doses or in overdose, LTG may exhibit non-linear pharmacokinetics due to saturation of metabolic pathways, particularly glucuronidation. This leads to a disproportionate rise in plasma concentration, prolonged elimination half-life, and an increased risk of toxicity [5]. Non-linear elimination occurs when drug clearance deviates from first-order kinetics, meaning elimination is no longer directly proportional to plasma concentration. Instead, saturation of metabolic pathways, such as CYP450 and UGT, results in zero-order kinetics, where a constant amount of drug is eliminated per unit time. Consequently, higher doses lead to a greater-than-expected increase in plasma levels and a prolonged half-life, heightening toxicity risk [6,7]. Clinically, careful dose adjustments and therapeutic drug monitoring are essential to prevent drug accumulation and toxicity. Sudden changes in clearance can lead to unexpected plasma level fluctuations, emphasizing the need for close monitoring [8].

Factors contributing to reduced LTG clearance include advanced age, elevated body weight, and interactions with concomitant medications. Drugs such as valproate or phenytoin may either compete for or enhance glucuronic conjugation, thereby increasing or decreasing LTG serum concentrations, respectively [9]. Despite a lack of consensus on the therapeutic range for LTG in terms of efficacy and safety, serum concentrations exceeding 20 mg/L have been associated with a need for dose adjustments or treatment discontinuation in approximately 25% of patients, suggesting a link between elevated concentrations and toxicity [10].

Alyahya et al. [3] reported that LTG toxicity is concentration-dependent, with interindividual variability playing a significant role. Interestingly, the severity of toxicity did not consistently correlate with the ingested dose. Publication bias may also influence the interpretation of case reports on LTG overdoses. Two fatal cases of massive LTG overdose further support the hypothesis of concentration-dependent toxicity. French et al. [11] described a 19-year-old male who ingested 4,000 mg of LTG. The patient developed seizures, refractory prolonged QRS complexes, complete heart block, cardiac arrest, aspiration pneumonia, diffuse encephalopathy, and infectious disseminated intravascular coagulation (DIC). Despite intensive care, the patient succumbed on day 10 following the dignified withdrawal from therapy. His LTG serum concentration was 35.7 mg/L, measured 19 hours after ingestion. Similarly, Nogar et al. [12] detailed a case involving a 48-year-old female who ingested 7,500 mg of LTG. She presented with seizures, refractory prolonged QRS complexes, pulseless cardiac arrest, and subsequent anoxic brain injury. She passed away on day four after therapy was withdrawn. Her LTG concentration reached 74.7 mg/L. In this case, LTG serum concentrations were measured at three time points as part of the patient's clinical monitoring. At the first point, 13 hours after ingestion, the concentration was at the upper limit of the normal range. This was unexpectedly lower than the predicted value calculated using previously reported pharmaceutical data based on a one-compartment linear first-order kinetic model, considering the drug's volume of distribution and clearance. At the second measurement, 55 hours after ingestion, the concentration was recalculated using an extended elimination half-life of 100 hours derived from the first sample. However, by the third measurement, at 79 hours after ingestion, the calculated elimination half-life aligned more closely with previously reported values.

Several reports document the time course of LTG serum concentrations through multiple assays following overdose. Although specific details, such as exact ingestion times, sampling intervals, and patient body weights, are often lacking, serum concentrations ranging from 26 to 69 mg/L have been observed in 2-5 assays conducted between days two and nine after ingestion of 3,500-40,000 mg of LTG. These studies provided limited discussion on the time course to elimination but estimated elimination half-lives ranging from 15 to 197 hours based on first-order kinetics. A general trend noted across cases is that elimination half-life tends to be prolonged early after ingestion, with gradual normalization over time (Table 4) [13-15].

**Table 4:** Estimated elimination half-life in previous reports These reports document the time course of LTG serum concentrations through multiple assays following overdose. Although specific details, such as exact ingestion times, sampling intervals, and patient body weights, are often lacking, serum concentrations ranging from 26 to 69 mg/L have been observed in 2-5 assays conducted between days two and nine after ingestion of 3,500-40,000 mg of LTG. These studies provided limited discussion on the time course to elimination but estimated elimination half-lives ranging from 15 to 197 hours based on first-order kinetics. A general trend noted across cases is that elimination half-life tends to be prolonged early after ingestion, with gradual normalization over time. Abbreviations: conc.: Concentration, LTG: Lamotrigine, NA: Not available, yo: years old †t1/2 = ln2 / kel, kel = ln (Cmax /Cmin) / Δ(t); t1/2 elimination half-life, kel elimination rate constant

Author	Year published	Age (years)	Body weight	Amount of LTG	Periods after ingestion	LTG concentration	Estimated elimination half-life^†^	Outcome
Sirianni et al. [13]	2008	17	55 kg	4,000 mg	6.5 hours	26 mg/L		Alive
					12.75 hours	24 mg/L	54 hours	
					18.25 hours	21 mg/L	29 hours	
					28.75 hours	18 mg/L	47 hours	
Sbei et al. [14]	2001	55	NA	NA	1 day	32 mg/L		Alive
					5 days	15 mg/L	88 hours	
Hajiali et al. [15]	2015	26	NA	40,000 mg	1 day	69 mg/L		Alive
					2 days	73 mg/L		
					3 days	58 mg/L	72 hours	
					5 days	49 mg/L	197 hours	
					9 days	28 mg/L	119 hours	

The delayed metabolism of LTG can be attributed to several factors. First, drug-drug interactions, such as those with valproic acid, a strong UDP-glucuronosyltransferase (UGT) inhibitor, non-steroidal anti-inflammatory drugs (NSAIDs), and oral contraceptives, can slow LTG clearance by inhibiting UGT enzymes. Liver dysfunction, such as cirrhosis or hepatitis, can impair glucuronidation, further delaying metabolism [16]. Genetic polymorphisms in UGT enzymes can lead to reduced enzyme activity, especially in poor metabolizers, causing higher drug levels. Age-related factors, such as immature UGT activity in neonates or reduced liver function in the elderly, can also impact LTG metabolism [17]. Pregnancy increases UGT activity, speeding up metabolism, but a drop in enzyme activity postpartum may result in delayed metabolism. Renal dysfunction may indirectly affect LTG metabolism due to impaired clearance of metabolites. A clinical case suggests that co-ingested acetaminophen could have caused liver dysfunction and impaired glucuronidation, requiring N-acetylcysteine treatment despite normal liver enzyme levels. Non-linear pharmacokinetics, such as delayed or saturated absorption from the digestive system, might also contribute to delayed metabolism, but renal dysfunction and age-related factors are less likely causes. Pregnancy was ruled out during the clinical course [18].

In our patient, the reasons for the initially lower concentration and the extended elimination half-life observed in the first evaluation remain unclear. Contributing factors such as young age, normal weight, and absence of liver disease were ruled out. Additionally, while olanzapine and risperidone were co-ingested, both drugs are metabolized via CYP450 pathways, which are not considered significant contributors to LTG metabolism or its interactions. Although there is no established consensus regarding the pharmacokinetics of LTG following an overdose, it is speculated that delayed LTG elimination results from the saturation of the glucuronidation pathway due to elevated LTG concentrations following massive ingestion. This saturation may lead to non-linear clearance, as supported by the observation that LTG elimination improved over time in parallel with the normalization of liver function tests.

There is currently no established treatment protocol for LTG overdose. While most reported cases involved gastric decontamination with activated charcoal and general supportive care, such as fluid therapy or oxygen supplementation, some patients required additional interventions, including intravenous lipid emulsion (ILE) therapy - leveraging the lipid sink theory for enhanced endogenous absorption - or extracorporeal removal via hemodialysis [13,19,20].

In our case, the clinical course aligned with previously reported outcomes. The patient exhibited no significant abnormalities in vital signs, electrocardiographic changes, or dermatological findings. The coma and mild elevation in liver enzymes were transient. She was discharged on day seven without residual deficits. Contributing factors to her relatively mild clinical course likely included her young age, absence of significant comorbidities, and only mildly elevated LTG peak serum concentration.

NAC, a glutathione-replenishing agent commonly used for acetaminophen-induced acute liver injury, was administered in this case. While some evidence supports NAC’s use in non-acetaminophen-induced acute liver failure (NAI-ALF) [21], its efficacy in LTG overdose remains undocumented. In our patient, the mild liver injury resolved following NAC therapy. However, since an acetaminophen overdose was also present, the specific contribution of NAC to the recovery of liver function in the context of LTG-induced NAI-ALF is unclear. Moreover, NAC did not appear to influence the rate of LTG elimination, as serial LTG concentrations demonstrated no significant change attributable to NAC administration.

## Conclusions

LTG is an antiepileptic that stabilizes neuronal membranes by inhibiting sodium channels. It is primarily metabolized in the liver via glucuronidation, with renal excretion of inactive metabolites. This report discusses a patient’s clinical presentation and LTG serum measurements following a significant overdose. Non-linear elimination with a prolonged half-life (~100 hours) was initially observed, normalizing by the third measurement, likely due to hepatic metabolism saturation. Age, BMI, and the absence of hepatic disease or drug interactions did not explain the delayed clearance. In severe LTG overdose, serial serum monitoring and kinetic assessment guide management. Interventions such as ILE therapy or dialysis may be considered. Prolonged consciousness disturbance in overdose cases may indicate metabolic dysfunction and an extended drug half-life, requiring further investigation and accurate blood drug concentration measurement to guide treatment.
